# Family-centered Care for Children with Disabilities in Japan—the Origin and Future of the “Ryouiku”

**DOI:** 10.1298/ptr.R0029

**Published:** 2024-03-12

**Authors:** Nobuaki HIMURO

**Affiliations:** ^1^Department of Public Health, School of Medicine, Sapporo Medical University, Japan

**Keywords:** Family-centered care, Pediatric physiotherapy, Glocal, Ryouiku

## Abstract

Family-centered care is recommended as the best practice in pediatric physiotherapy. It is a philosophy that pediatric physiotherapists should be familiar with, as it relates to the health of the child as well as the family. However, family-centered care has not been adequately taught in physiotherapy education in Japan. The term “Ryouiku” was coined in 1940 in Japan. The concept of the Ryouiku is essentially Evidence-Based Medicine and is the very essence of family-centered care with a focus on function. By re-thinking the Ryouiku and applying it to pediatric physiotherapy education in Japan, “glocal” family-centered care can be practiced in a Japanese setting.

## Introduction

Family-centered care is widely recommended as the best practice in pediatric physiotherapy^1–3)^. Family-centered care increases family as well as health-care professional satisfaction, improves health outcomes, uses the health-care resources more effectively, and decreases health-care costs^4)^.

Family-centered care is an established approach for delivering services in the context of children’s health care, mainly in Canada^4)^. There are many reports investigating the implementation of family-centered care internationally, including in Japan. However, the family-centered care definitions used in them are mostly based on Canadian studies. There are strong cultural influences in the family context, and the way family-centered care is adapted to each culture needs to be considered^5)^. In other words, it is important to think scientifically about family-centered care from a global perspective and practice clinical research locally. In this review, I would like to describe the “glocalization” of family-centered care in Japan.

## Family-centered Care

The family plays a central role in the child’s life. The core of all decision-making is the family, including their children^5)^. Family plays a vital role in promoting the health and well-being of their children. Family-centered care recognizes the family as the key element in achieving better outcomes in pediatric health care^4)^. Family-centered care includes the parents being the primary decision makers, treating them with respect and support, and providing them with the information they need^6)^. It is an attitude and approach toward the child and family, and a philosophy that physiotherapists should be equipped with^1,5)^.

Family-centered care refers to an approach to service delivery for children and parents that recognizes 1) parents are the experts on their child’s health, abilities, and needs; 2) every family is unique; and 3) optimal child functioning occurs within a supportive family and community context^5)^. Decision-making by the family, collaboration, mutual respect, support, individualized and flexible interventions, and sharing are important^4)^.

Family-centered care is associated with improved parental knowledge of child development, increased parental satisfaction, and improved parental mental health^1,3,7–9)^. Family-centered care promotes children’s development, skill acquisition, and psychological adjustment^6,10)^. Family-centered care is thus important for the health of both the child and the family. In addition, family-centered care is also associated with parents’ educational level, employment status^11)^, and family income^12)^, as well as the experience and training of the health-care provider^13)^.

## The Measure of Processes of Care to Measure Family-centered Care

Rosenbaum et al. developed a conceptual framework of family-centered care^5)^, along with a tool to measure parents’ experiences with the delivery of health services called the Measure of Processes of Care (MPOC)^14)^. MPOC has been adopted for both research and practice evaluation internationally^15)^.

The MPOC is a self-reported questionnaire that measures how parents think about the care their children are provided^14)^. In other words, it evaluates the family-centered attitude and behavior of the health-care provider. Responses are made using a seven-point scale. Response options range from 1 (never) to 7 (to a great extent). The MPOC includes five scales: Enabling and Partnership, Providing General Information, Providing Specific Information about the Child, Coordinated and Comprehensive Care, and Respectful and Supportive Care. The average score is calculated for each scale. A shortened version (MPOC-20) with 20 questions has also been developed^16)^.

In a systematic review and meta-analysis of the study on the MPOC-20^17)^, the Providing General Information scores were found to be subtractive compared to other scales. Service providers are encouraged to focus on child and family needs for general information. There are several reports investigating the implementation of family-centered care using the MPOC in East Asia^8,18–20)^ (Table 1). Similar to Canada, it can be seen that family-centered care in East Asian countries tends to score higher on Respectful and Supportive Care and lower on Providing General Information. The Japanese version of the MPOC was evaluated with 261 parents of children with disabilities^8)^ and, as in other countries, the results were low in terms of providing information. Communication styles may be particularly susceptible to cultural influences. The way information is provided is an interesting subject for future research.

**Table 1.** The measure of processes of care scores in Canada and East Asia

Study	Country	Enabling and partnership, mean (SD)	Providing general information, mean (SD)	Providing specific information about the child, mean (SD)	Coordinated and comprehensive care, mean (SD)	Respectful and supportive care, mean (SD)
King et al.^16)^	Canada	5.10 (1.55)	4.09 (1.77)	5.23 (1.48)	5.25 (1.39)	5.40 (1.29)
Himuro et al.^8)^	Japan	5.43 (1.27)	4.09 (1.63)	5.30 (1.50)	5.46 (1.26)	5.66 (1.18)
Wang et al.^18)^	China	5.09 (0.86)	4.62 (0.85)	4.90 (1.10)	5.05 (0.97)	5.26 (0.84)
An et al.^19)^	Korea	5.31 (1.36)	4.25 (1.71)	4.45 (1.77)	5.22 (1.44)	5.45 (1.34)
Tew and Ahmad Fauzi^20)^	Malaysia	5.65 (0.93)	4.79 (1.29)	4.50 (1.51)	–	5.60 (1.09)

SD, standard deviation

MPOC has been in development for more than a quarter century. The developers of the original MPOC, knowing what today’s parents want, need, and expect from healthcare providers, have set out to develop a new version for the times. New components of care that go beyond what was identified in the original MPOC include effective communication, practical support (in addition to emotional and informational support), and availability and scheduling^21)^. The results of this study also suggest that communication between families and healthcare providers will be an important issue in the future.

## Issues for the Family-centered Care in Japan

Although family-centered care was described as the ideal model for pediatrics, implementation of family-centered care was reported to be difficult. Parents and providers have reported that many services are not sufficiently family-centered^17,21–23)^. There is a knowledge translation gap between theory and practice, and one reason is inadequate training of health-care providers^22,24)^.

Experimental and reflective activities are keys to promoting family-centered care^25)^. Although there are some reports from Western countries regarding the incorporation of patient- and family-centered care training into curricula of medical education, these curricula and/or programs have not yet integrated family-centered care in Japan. As the importance of family-centered care training has been reported in non-Western countries^26)^, the curriculum should be considered in medical education in Japan.

## The Origins of the Ryouiku

What does it mean to think locally about family-centered care in Japan? The uniquely Japanese concept of children with disabilities cannot be ignored. This is, the “Ryouiku.”

The term Rouiku was coined in the 1940s by Kenji Takagi, the second professor of the Department of Orthopedics at the then Tokyo Imperial University (now the University of Tokyo)^27)^. In essence, this means that using every modern science we can make the best use of children’s abilities. It also has overtones of allowing children to be self-sufficient. He insisted on the importance of the so-called trinity of medical care, education, and job training at the same time.

Two individuals were important influences in Takagi’s creation of the concept of the Ryouiku^28)^. One is Yoshinori Tashiro, and the other is Matsuzou Kashiwakura.

In the 1910s and 1920s, when Takagi was a member of the medical staff of the Department of Orthopedics at Tokyo Imperial University, Tashiro, the first professor of the Department of Orthopedics, appealed for a survey of the actual conditions of the children with physical disabilities after returning from a tour of Europe. This had a significant impact on Takagi^27)^. Takagi conducted a survey of children with disabilities after much difficulty and revealed that many children with disabilities were living in terrible conditions in the slums of Tokyo at the time, hidden away. He also conducted a survey of school children, which revealed that many children with disabilities were among those who did not come to school. The fact that many children with disabilities who had a chance of recovery did not have the opportunity to receive treatment, and that there were many children who could not receive education because of their disabilities, led him to envision the establishment of an institution where they could receive education while receiving treatment.

At the same time, Kashiwakura, a member of the Department of Orthopedics, established a school where students could receive what is now called rehabilitation while receiving an education, which may have influenced Takagi. Kashiwakura was formerly a physical education teacher and a pioneering physiotherapist who designed and provided exercises for children with physical disability^29)^.

The idea of Tashiro, Kashiwakura, and Takagi was “to give children with disabilities a way to live on their own”^28)^. Although they shared a common view that this would require treatment and education, they took different paths due to differences in their specific goals. Tashiro aimed to establish an public school for children with physical disabilities, Kashiwakura opened his own academy to provide education and physiotherapy to children with physical disabilities, and Takagi went on to establish a facility for children with physical disabilities that was mainly for medical care.

Takagi also referred to the concrete intervention for children with cerebral palsy. A child with cerebral palsy, who could not use a spoon and was not independent in eating, was able to turn the dial on a radio, a valuable commodity at the time, to produce sound. Inspired by this, he replaced the dial with the shaft of a fountain pen, and the child was able to write six months later. Based on this episode, in the 1950s, as the basis for intervention for children with cerebral palsy, he emphasized that play itself, which is an unintentional passion, should be medical treatment, and that coercion or reprimanding would not motivate the child to continue^30)^.

## Transitions in the Ryouiku

The term Ryouiku was used to describe the combination of education and vocational training based on orthopedic medical treatment to enable social participation. It did not cover, for example, intellectual disabilities or severe multiple disabilities. However, this limitation is largely due to the historical circumstances of the time, when children with hip dislocation and polio were excluded from education and occupation despite their great potential for social participation. Takagi seemed to think that there was another way to help children with severe cerebral palsy. Since the 1960s, legislation for institutions for children with severe multiple disabilities has been in place. Today, the term has come to describe the entire approach to children with disabilities that has continued since childhood. This is because the social involvement of children with disabilities is changing from moment to moment.

Since the 1970s, attention has focused on early detection and early rehabilitation for cerebral palsy, and a Ryouiku system has been developed in Japan. Around this time, the Boberth and Vojita methods were introduced to Japan, and physiotherapists began intervening actively to improve motor function. This was aimed at improving the quality of movement (i.e., bringing it closer to normal), which means that a different approach may be used than the one that Takagi had based his intervention for cerebral palsy on.

In the 1980s, Takamatsu, a pediatric orthopedic surgeon, described in his book^31)^ the Ryoiuku as the basis for approaching all children with disabilities, writing that “Ryouiku is an emotion, an idea, a science, and a system.” Emotion for the child and family is important. Physiotherapy for children cannot be established by a collection of techniques alone; a deep thought is important, and it must be scientific. It is a team approach, not one that relies solely on individual efforts, and a system that includes involvement over time with age-related changes is important. The Ryouiku is an attempt to expand the freedom of children with disabilities using all current science and civilization, which must be excellent “parenting”^31)^. In other words, it may be said that the Ryouiku was conceptualized as including the modern concepts of Evidence-Based Medicine^32)^ and family-centered care.

## Re-thinking Ryouiku for Family-centered Care in Japan

In recent years in Japan, the term the Ryouiku has been used as a kind of methodology for early intervention for children with developmental disabilities. In other words, it is being used in a manner different from the original concept of the Ryouiku that Takagi and his colleagues had in mind. Unfortunately, few physiotherapists in Japan practice family-centered care on a scientific basis. It is important to consider the concept of family-centered care in Japan by taking a fresh look at the history of Ryouiku. The Ryouiku is essentially the Evidence-Based Medicine, a concept that also includes emotion as a new perspective related to family-centered care. Furthermore, the Ryouiku is a function-focused intervention recommended worldwide^33)^.

Family involvement in pediatric physiotherapy in Japan, which has been practiced through accumulated experience, is effective for the child and family. Think about the family-centered care from a global perspective and put it into practice locally. To achieve this, pediatric physiotherapy in Japan is required to develop a “glocal” pediatric physiotherapy based on the latest science, based on the concept of the Ryouiku that originally existed in Japan. I believe that relearning the concept of the Ryouiku is necessary for the practice of “glocal” family-centered care in Japan.

## Conflict of Interest

The author declares no conflicts of interest.
